# Extended crop yield meta-analysis data do not support upward SCC revision

**DOI:** 10.1038/s41598-025-90254-2

**Published:** 2025-02-15

**Authors:** Ross McKitrick

**Affiliations:** Department of Economics and Finance, University of Guelph, Guelph, Canada

**Keywords:** Climate change, Agricultural yields, Social cost of carbon, Carbon dioxide fertilization, Climate-change impacts, Climate-change policy, Environmental health

## Abstract

The Biden Administration raised its Social Cost of Carbon (SCC) estimate about fivefold based in part on global crop yield decline projections estimated on a meta-analysis data base first published in 2014. The data set contains 1722 records but half were missing at least one variable (usually the change in CO_2_) so only 862 were available for multivariate regression modeling. By re-examining the underlying sources I was able to recover 360 records and increase the sample size to 1222. Reanalysis on the larger data set yields very different results. While the original smaller data set implies yield declines of all crop types even at low levels of warming, on the full data set global average yield changes are zero or positive even out to 5 °C warming.

## Introduction

Recent estimates of the Social Cost of Carbon (SCC) from the US Environmental Protection Agency (EPA)^1^ are about 5 times higher than previously^2^. Part of the increase is due to an upward revision of the estimated agricultural damages from climate warming. The EPA used two damage modules, denoted DSCIM and GIVE, and in the latter, of the new 2030 SCC value ($220 under 2% discounting), $103, or nearly half, is attributed to projected agricultural damages (^1^ pp. 78–81). The GIVE agricultural damage function is based on^3^ (Moore et al. 2017, herein “M17”) which presents a reanalysis of a database first presented in^4^ (Challinor et al. 2014, herein denoted “C14”), which itself was a meta-analysis of crop model studies simulating yield changes for agricultural crops under various climate warming scenarios. The underlying models were parameterized based on results from field studies, and the authors selected them to be, as much as possible, globally representative.

In their multivariate model C14 reported results that would imply a moderate global net benefit from the warming associated with doubling the atmospheric CO_2_ level. Despite using the same data, M17 reached much more pessimistic conclusions, projecting declining global crop yields due to the warming. Since both studies only provided limited reporting of regression results and the models are not nested it is difficult for readers to trace the sources of the differences. The first purpose of this study is to provide a transparent analysis of the C14 dataset, reproducing both sets of results as closely as possible, then to examine the effects of extending the underlying data set.

The C14 dataset consisted of 1722 records but only half (*N* = 862) had complete observations of all the variables necessary for regression analysis (changes in yield, CO_2_ levels, temperature and precipitation matched to information about the climate zone, adaptation efforts and crop type). The variable most commonly missing was the change in ambient CO_2_. But re-examination of the underlying sources showed that in many cases these could be recovered, for instance by consulting the original climate scenario tables. It was thus possible to increase the usable sample size by 40% to *N* = 1222. M17 critiqued the regression specification in C14 and estimated a model with many more interaction terms, though most were insignificant. The carbon dioxide fertilization benefit also differs between the two studies, with C14 treating it as linear and M17 imposing concavity (diminishing marginal gains).

Herein I replicate the results in C14 and M17 on the (incomplete) C14 dataset and I also obtain simulation results that qualitatively support the findings in M17 of negative yield effects across crop types especially soybeans. But after incorporating the newly-available data the conclusions change such that global average yield gains of all crop types under CO_2_-induced warming are positive even out to 5 °C warming. Overall I conclude that the climate change-related agricultural damage estimates in M17 are too pessimistic and the large implied revisions to the SCC are unsupported.

## Background

**Fig. 1:**
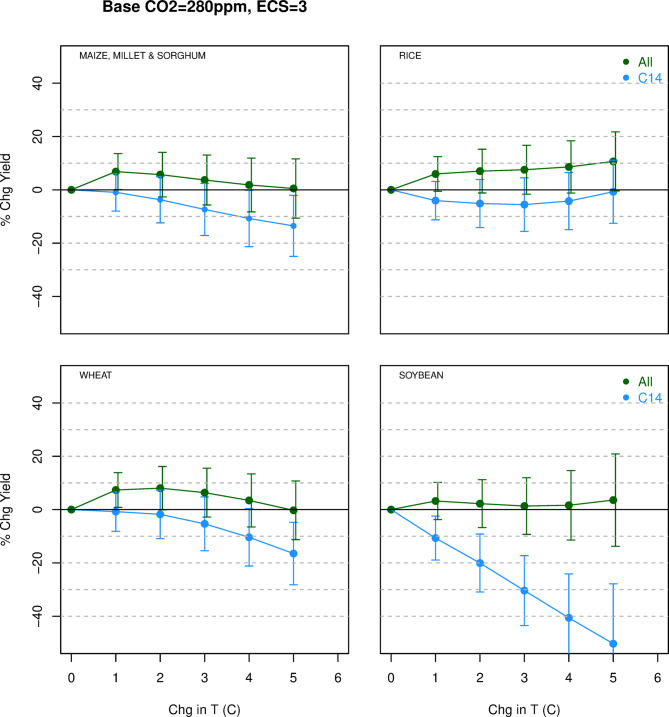
Yield change simulations based on indicated data sets. Blue: C14 data. Green: All data.

The main climatic influences on agricultural yields are increased ambient CO_2_ levels (positive effect), higher temperatures (mix of positive and negative effects) and changed precipitation (mix of positive and negative effects). Based on laboratory and controlled field studies the literature has generated a profusion of yield change estimates that vary by region, plant type, adaptation efforts and other covariates^5^. For the purpose of computing the SCC we are only interested in the net effect of warming caused by increased CO_2_, not of warming alone. Both M17 and C14 present bivariate graphs of partial yield effects from temperature change alone which can be misleading in the anthropogenic climate change context. For example M17 Fig. 1 shows temperature effects on yields of four major crop types excluding the offsetting effect of CO_2_ fertilization and adaptation. In all cases the effects are negative, but by construction such graphs only illustrate the effects of unanticipated natural warming trends not CO_2_-induced warming.

Table 1 reports summary statistics of both the C14 dataset and the expanded version developed herein, columns denoted respectively as “C14” and “All”. When analyzing CO_2_-induced warming any assumed temperature change (and induced precipitation change) must be based on an associated CO_2_ change, the computation of which requires an assumption about climate sensitivity. For agricultural yield simulations in which CO_2_ and temperature levels change gradually together a common metric is the Transient Climate Response (TCR) which estimates the temperature change at year 70 in a simulation in which CO_2_ levels increase by 1% per year, which implies doubling at year 70. The most recent IPCC report^6^ provides a best estimate of TCR based on observed temperature and ocean heat content records of 1.9 °C. Since atmospheric CO_2_ levels are growing at just under 0.6% per year since 1990^7^ doubling will take about 120 years. An alternative metric is Equilibrium Climate Sensitivity (ECS) which is the eventual (multi-century) temperature response after all Earth systems including the oceans have adjusted to an instantaneous CO_2_ doubling. It has traditionally been estimated at about 3 °C. While TCR is more relevant to the simulations herein, I will conservatively employ a 3 °C sensitivity estimate which implies less CO_2_ increase for a given temperature change.

**Table 1:** Summary statistics of C14 dataset and extended (“All”) data set.

	Mean	Med	Std dev	Min	Max
C14 (*N* = 862)					
Chg in yield (%)	− 4.82	− 4.00	20.30	− 81.80	62.30
Chg in temperature (°C)	2.72	2.42	1.37	0.00	8.67
Chg in precip (mm)	6.50	6.00	16.76	− 46.00	194.00
Chg in CO_2_ (ppm)	161.23	192.50	137.66	0.00	504.50
Tropics	0.52	1.00	0.50	0.00	1.00
Adaptation	0.40	0.00	0.49	0.00	1.00
C4	0.34	0.00	0.47	0.00	1.00
Baseline temp (°C)	20.95	21.25	5.17	10.10	27.69
ALL (*N* = 1222)					
Chg in yield (%)	− 3.81	− 4.00	23.11	− 90.50	135.00
Chg in temperature (°C)	2.67	2.43	1.39	− 1.21	8.67
Chg in precip (mm)	5.50	5.00	16.16	− 46.00	194.00
Chg in CO_2_ (ppm)	176.18	202.00	132.94	0.00	504.50
Tropics	0.46	0.00	0.50	0.00	1.00
Adaptation	0.38	0.00	0.49	0.00	1.00
C4	0.32	0.00	0.46	0.00	1.00
Baseline temp (°C)	20.83	21.25	5.55	7.85	27.69

The standard, stylized physics of warming^8,9^ is summarized as a logarithmic relationship between the change in temperature ($\Delta T$) and the log CO_2_ increase such that $\Delta {T}_{\tau }=\alpha {\lambda }^{-1}\text{ln}\left(\frac{C{O}_{2}(\tau )}{C{O}_{2}(0)}\right)$ where $\tau$ denotes the interval in years since time 0, $\Delta {T}_{\tau }$ denotes the amount of warming over that interval, $C{O}_{2}(\tau )$ denotes the ambient CO_2_ concentration at time $\tau$, ${CO}_{2}(0)$ denotes the ambient CO_2_ concentration at time 0, $\alpha$ parameterizes the relationship between log CO_2_ and radiative forcing and ${\lambda }^{-1}$ parameterizes the relationship between radiative forcing and global average temperature. For the present purpose we do not need to separately identify $\alpha$ and $\lambda$ instead we can compute them jointly using $\alpha {\lambda }^{-1}=\text{ECS}/\text{ln}(2)$. The change in CO_2_ required for a given temperature increase $\Delta T$ is then given by

1 $${\Delta C}_{\uptau }\equiv C{O}_{2}\left(\tau \right)-C{O}_{2}\left(0\right)=C{O}_{2}\left(0\right)\times \left(\text{exp}\left\{\frac{\Delta {T}_{\tau }}{\alpha {\lambda }^{-1}}\right\}-1\right).$$

The results shown in Fig. 1 are not overly sensitive to the year selected as the baseline. I will conservatively assume time zero corresponds to preindustrial conditions hence $C{O}_{2}(0)=280$ ppm^10^. Thus we have in mind an ongoing scenario with approximately 1 °C of warming having taken place already. A key implication of the standard model is that a linear increase in temperature requires an exponential increase in CO_2_: if for example 100 ppm yields 1°C warming, the next 1°C warming requires 200 ppm, then 400 ppm, etc.

On their dataset C14 estimated the linear regression model

2 $$d{Y}_{i}={\alpha }_{0}+{\alpha }_{1}A{D}_{i}+{\alpha }_{2}T{R}_{i}+{\alpha }_{3}C{4}_{i}+{\alpha }_{4}d{P}_{i}+{\alpha }_{5}d{T}_{i}+{\alpha }_{6}d{C}_{i}+{e}_{i}$$

where $d{Y}_{i}$ is the % change in yield for region *i*, $A{D}_{i}$ is a dummy variable denoting that the study incorporated adaptative behaviour, $T{R}_{i}$ is a dummy variable denoting the region is in the tropics as opposed to a temperate zone, $C{4}_{i}$ is a dummy variable indicating that the crop type in the study is C4 (maize, millet or sorghum) rather than C3 (rice, wheat, soybeans and other crops), (The labels C3 and C4 refer to the photosynthesis process, specifically the carbon compounds produced within the plant.) $d{P}_{i}$ denotes change in precipitation, $d{T}_{i}$ denotes change in temperature, $d{C}_{i}$ denotes the change in the CO_2_ concentration and ${e}_{i}$ is the regression residual. The coefficient estimates in C14 (see Table 2 below) imply a partial temperature effect on yield of − 4.9% per °C and a partial CO_2_ effect of + 0.06% per part per million (ppm), both of which were reported to be highly significant (*p* < 0.01). These coefficients imply that if the atmospheric concentration of CO_2_ doubles from 280 to 560 ppm and causes 3 °C warming, the combined effect on yields would be, on average, − 14.7% (due to warming) plus 16.8% (due to CO_2_ fertilization) for a net effect of + 2.1%.

**Table 2:** Columns 1 and 2: comparison of temperature- and precipitation-only yield changes computed by M17 (column 1) and the same computed herein on the C14 dataset as supplied using the method described in the text (column 2). Columns 3 and 4: point estimates of yield changes (%) estimated on C14 dataset (column 3) and expanded data set (“All”, column 4).

	M17 result	Replication on C14 data	C14	All
Maize, millet and sorghum				
dY = 1C	− 6.1	− 7.9	− 0.9	6.8
2C	− 14.0	− 14.9	− 3.7	5.7
3C	− 23.7	− 21.0	− 7.4	3.7
4C	− 5.6	− 9.4	− 10.7	1.8
5C	− 10.3	− 16.1	− 13.5	0.5
Rice				
dY = 1C	− 14.1	− 20.1	− 4.0	5.9
2C	− 4.0	− 6.1	− 5.1	7.0
3C	− 10.5	− 12.8	− 5.5	7.5
4C	− 19.3	− 19.9	− 4.2	8.6
5C	− 14.2	− 16.1	− 0.7	10.6
Wheat				
dY = 1C	− 26.4	− 31.0	− 0.8	7.3
2C	− 36.7	− 44.9	− 1.8	8.0
3C			− 5.3	6.4
4C			− 10.4	3.4
5C			− 16.5	− 0.3
Soybean				
dY = 1C			− 10.7	3.2
2C			− 20.0	2.2
3C			− 30.4	1.3
4C			− 40.6	1.6
5C			− 50.3	3.6

C14 included $d{C}_{i}$ as a linear term in their estimating equation, which implies that marginal benefits of CO_2_ fertilization do not attenuate. By contrast M17 used a concave function

3 $$fC\left(d{C}_{i},C{4}_{i}\right)=\frac{d{C}_{i}}{A+\left(1-C{4}_{i}\right)B+d{C}_{i}}$$

with *A* and *B* chosen to equal 50. This dampens the marginal benefit of additional CO_2_. M17 (Supplement) report a CO_2_ fertilization benefit of only 8–12% from CO_2_ doubling depending on crop type. Another methodological difference introduced by M17 was to argue that the regression specification should restrict yield changes to zero if no climate change takes place ($$d{T}_{i}=d{P}_{i}=d{C}_{i}=0)$$. In the specification in C14, adaptation without climate change would generate a gain of about 7% in the temperate zone and about 4% in the tropics, which indicates that the constant and dummy terms in Eq. (2) are not strictly measuring variables of interest for estimating the SCC. M17 included adaptation as an interaction with climate variables, however they also included the adaptation dummy on its own to measure yield gains due to non-climate-related adaptation activity, which they then subtracted back out from the predicted yield changes, an approach I also use herein.

## Results

Yield change predictions for each crop type were generated using the slope coefficient estimates from replication regressions (see “Methods” section) conditioned on sequential values of $dT$ from 1.0 to 5.0 and the corresponding changes in CO_2_ fertilization from Eq. (1) and precipitation. To project the warming-induced change in precipitation, $d{P}_{i}$ was regressed on $d{T}_{i}$, $d{T}_{i}^{2}$ and $d{T}_{i}$ interacted with national baseline temperatures with no intercept, separately by crop type. The coefficients were then used to generate $d{\widehat{P}}_{i}$ conditioned on the assumed value of $dT$ and the mean baseline temperatures for each region. $2\sigma$ error bars were computed using bootstrap resampling with 1,000 replications.

Table 2 columns 1 and 2 compare the projected yield changes for 1–3 °C warming as computed by M17 suppressing CO_2_ fertilization and adaptation (F. Moore pers. comm.) and the same computed using the method herein on the C14 dataset. While the results are not identical the columns are sufficiently similar (correlation = 0.96) to establish the validity of the replication.

Yield change estimates for warming of 1 °C to 5 °C (in %) based on the C14 and “All” datasets taking account of CO_2_ fertilization and adaptation are shown in Table 2 columns 3 and 4 and Fig. 1. Rice, wheat and soybean were simulated separately. Figure 1 shows the results with the lines labeled, respectively, “C14” (blue) and “All” (green).

M17 Supplementary Figs. 2–5 show regional yield changes are a mix of positive and negative globally at 1 °C for maize, rice and wheat but go negative almost everywhere by 3 °C. For soybeans yield changes are globally negative even at 1 °C and rapidly worsen from there. The replications herein (blue lines in Fig. 1) match these expectations. But adding in the missing data noticeably changes the results. We now observe insignificant but positive average output gains for all crop types across the warming scenarios even up to 5 °C (at 5 °C wheat drops slightly below zero). The negative temperature effects are fully offset by gains from CO_2_ fertilization and adaptation. In the Supplement I show that virtually identical results are obtained using any configuration of the two key parameters, climate sensitivity (1.9°C or 3.0°C) and the CO_2_ base case level (280 ppm or 370 ppm).

## Discussion

In a climate change scenario relevant to policymaking temperature changes in response to CO_2_ increases, and precipitation changes in response to temperature changes. Consequently the analysis needs to be done using multivariate modeling, which unfortunately disqualified half the C14 dataset. On that version of the dataset I replicated the regression results of C14 and generated regression results and yield change simulations approximately matching those in M17. But after rebuilding and extending the dataset I find different and much more optimistic results, namely that net crop yield changes are zero or positive even out to 5 °C for all crop types, even soybean.

I focus herein on global average outcomes which are the relevant ones for computing the SCC. Dividing crops by zone shows that warming in tropical regions is more harmful to crops than warming in temperate regions (results shown in Supplement). But climate change simulations, including in M17, generally predict relatively greater warming in temperate regions compared to the tropics. Since relatively less warming happens where it is relatively more harmful and vice-versa, the global average yield change remains a suitable and informative metric for assessing global outcomes even considering regional variations in outcomes.

The welfare changes in M17 are not based solely on the yield equations, but on feeding the agricultural output changes into a global computable general equilibrium model called GTAP. When output of a crop increases it can generate welfare reductions in some regions based on terms of trade effects. An increase in global agricultural productivity means exporting regions might lose revenue if the price falls by enough, while importing regions benefit and the net effect will be positive. If over the next 100–200 years yields of all crop types increase it does not stand to reason that a global trade model could generate global welfare reductions. Consequently the large global welfare losses associated with agricultural damages under climate warming as presented in M17 are not supported by the analysis of a more complete version of the crop yield meta-analysis data base. Neither therefore are the recent upward SCC revisions by the US EPA^1^ that derive from M17. The revisions that derive from other sources will need to be examined separately.

## Methods

I obtained the C14 dataset from A. Challinor (personal communication) the baseline ambient temperatures as used in M17 from Thomas Hertel (personal communication) and the M17 regression results and some simulation data from F. Moore (personal communication). C14 report $N=882$ complete records but I found only 862 in the data set as supplied, so while I refer to it as the “C14” dataset the results are not identical to those they reported. In the Supplement I describe the process by which an additional 360 entries were obtained. In all cases where the change in ambient CO_2_ was the only missing variable the underlying paper was re-examined. Often the name of the climate change scenario being used as an input into the crop model was given and the start and end dates of the model experiment were also given so the change in CO_2_ could be recovered by consulting past IPCC reports where the scenarios were developed. Where many or most explanatory variables were missing in the C14 dataset these were not re-examined or the attempt to recover the missing data was unsuccessful. Data and code sufficient to reproduce all reported results are in the data archive listed below the references.

Table 3 lists the regression results reported in C14 (column 1), the results of estimating Eq. (2) on the C14 dataset as supplied (column 2) and the results on the extended data (“All”, column 3). The estimations were done using R version 4.2.3^11^ and the *lm_robust* package^12^ clustering errors on the Study variable. *P-*values are shown in parentheses below the coefficients. In columns 2 and 3 they are computed using *t*-statistics based on robust standard errors. Adaptation and CO_2_ fertilization are clearly beneficial while temperature has a negative effect. Precipitation is significant only in the C14 reported results. The coefficient magnitudes are reasonably similar across the columns although significance levels are lower in the replication.

**Table 3:** Coefficients from estimation of equation^1^ on C14 dataset and expanded data set (“All”) compared to results reported in C14 (column 1). *p*-values of robust *t*-statistics in parentheses. Significance: < * 10%, ** 5%, *** 1%.

	C14 reported results	C14 data	All data
Intercept	− 5.40	− 5.67	− 3.60
	(0.44)	(0.63)	(0.71)
Adaptation	7.16**	7.42*	7.17**
	(0.02)	(0.06)	(0.04)
Tropics	− 2.83	− 4.09	− 3.36
	(0.47)	(0.43)	(0.48)
C4	0.00	− 0.02	− 2.27
	(0.99)	(0.99)	(0.59)
dPrecip	0.53***	0.25	0.26
	(0.00)	(0.35)	(0.14)
dTemp	− 4.90***	− 4.20**	− 4.69***
	(0.00)	(0.02)	(0.01)
dCO_2_	0.06***	0.06*	0.06*
	(0.00)	(0.10)	(0.07)
R-sq	NA	0.21	0.172
Adj R-sq	NA	0.21	0.168
F-stat	NA	2.44**	2.920**
N	882	862	1,222

M17 estimated a panel regression model with crop-specific quadratic temperature terms:

4 $$\begin{aligned} dY_{i} & = a_{1} MZ_{i} dT_{i} + a_{2} MZ_{i} dT_{i}^{2} + a_{3} RC_{i} dT_{i} + a_{4} RC_{i} dT_{i}^{2} + a_{5} WT_{i} dT_{i} + a_{6} WT_{i} dT_{i}^{2} \\ & \quad + a_{7} SB_{i} dT_{i} + a_{8} SB_{i} dT_{i}^{2} + a_{9} MZ_{i} dT_{i} BT_{i} + a_{{10}} MZ_{i} dT_{i}^{2} BT_{i} + a_{{11}} RC_{i} dT_{i} BT_{i} + a_{{12}} RC_{i} dT_{i}^{2} BT_{i} \\ & \quad + a_{{13}} WT_{i} dT_{i} BT_{i} + a_{{14}} WT_{i} dT_{i}^{2} BT_{i} + a_{{15}} SB_{i} dT_{i} BT_{i} + a_{{16}} SB_{i} dT_{i}^{2} BT_{i} + a_{{17}} C3_{i} \widehat{{fC}}_{i} \\ & \quad + a_{{18}} C4_{i} \widehat{{fC}}_{i} + a_{{19}} dP_{i} + a_{{20}} AD_{i} dT_{i} + a_{{21}} AD_{i} + e_{i} \\ \end{aligned}$$

where $M{Z}_{i}$, $R{C}_{i}$, $W{T}_{i}$ and $S{B}_{i}$ are dummy variables for, respectively, maize, rice, wheat and soybean, $B{T}_{i}$ is the average national baseline temperature (in °C) and ${\widehat{fC}}_{i}$ are the predicted values from Eq. (3) above with $A=B=50$. M17 estimated Eq. (4) using ordinary least squares (OLS) and obtained standard errors using the block bootstrap with the blocks defined at the study level. The first column of Table 4 reports the coefficient point estimates obtained by M17 M17 (*p*-values were not supplied). Column 2 (“C14”) reports the results of estimating Eq. (4) on the supplied version of the C14 dataset using OLS with cluster-robust errors. Column 3 (“All”) reports the results using the extended data set. Maize was taken to include millet and sorghum though there were only 2 observations of each of these. Columns 1 and 2 exhibit considerable similarity. Column 2 shows very few coefficients are statistically significant, although the regression itself is highly significant. The coefficients associated with soybean (“sb”) change considerably between the C14 and All datasets. The others remain more stable (correlation = 0.72).

**Table 4:** Column 1: estimated coefficients from F. Moore (pers. comm.) and M17 Supplement. Columns 2 and 3: coefficients from estimation of equation M17 on C14 dataset and expanded data set (“All”). *p*-values of robust *t*-statistics in parentheses. Significance: < * 10%, ** 5%, *** 1%.

	M17 coefs	C14 data	All data
mz.dT	3.714	2.635	5.490
		(0.882)	(0.662)
mz.dT2	− 0.887	0.165	− 1.613
		(0.967)	(0.596)
rc.dT	50.374	45.240*	23.450
		(0.093)	(0.523)
rc.dT2	− 12.778	− 11.819*	− 6.397
		(0.071)	(0.479)
wt.dT	− 5.595	− 6.672	− 3.068
		(0.618)	(0.751)
wt.dT2	1.871	2.676	0.665
		(0.335)	(0.815)
sb.dT	− 144.926	− 107.181	42.830
		(0.278)	(0.664)
sb.dT2	61.710	41.417	0.735
		(0.364)	(0.975)
mz.dT.bt	− 0.403	− 0.532	− 0.625
		(0.404)	(0.183)
mz.dT2.bt	0.038	0.013	0.089
		(0.942)	(0.539)
rc.dT.bt	− 2.223	− 2.140*	− 1.247
		(0.057)	(0.372)
rc.dT2.bt	0.521	0.499*	0.272
		(0.058)	(0.438)
wt.dT.bt	0.161	0.064	− 0.219
		(0.902)	(0.647)
wt.dT2.bt	− 0.180	− 0.199	− 0.062
		(0.166)	(0.693)
sb.dT.bt	5.818	4.075	− 2.570
		(0.332)	(0.550)
sb.dT2.bt	− 2.723	− 1.839	0.008
		(0.371)	(0.993)
c3.fC	17.20	23.929*	25.254**
		(0.061)	(0.027)
c4.fC	10.82	19.708	18.900
		(0.153)	(0.082)
dP	0.21	0.211	0.210*
		(0.243)	(0.087)
ad.dT	0.17	0.799	2.655
		(0.683)	(0.178)
ad	NA	5.487	− 0.901
		(0.263)	(0.850)
R-sq		0.418	0.399
Adj-R-sq		0.404	0.388
F		6749.509	568.225
N		862	1222

## Supplementary Information

Supplementary Information.

## Data Availability

Data and Code archive for this paper: https://data.mendeley.com/datasets/w6rpyk3gyc/1.

## References

[1] US Environmental Protection Agency. Supplementary Material for the Regulatory Impact Analysis for the Final Rulemaking, “Standards of Performance for New, Reconstructed, and Modified Sources and Emissions Guidelines for Existing Sources: Oil and Natural Gas Sector Climate Review”, EPA Report on the Social Cost of Greenhouse Gases: Estimates Incorporating Recent Scientific Advances, November 2023 (2023). https://www.epa.gov/system/files/documents/2023-12/epa_scghg_2023_report_final.pdf accessed February 28, 2024.

[2] US Interagency Working Group on Social Cost of Carbon (IWG). Technical Support Document: Technical update of the social cost of carbon for regulatory impact analysis Under Executive Order 12866. United States Government (2013).

[3] Moore, F. C., Baldos, U., Hertel, T. & Diaz, D. New science of climate change impacts on agriculture implies higher social cost of carbon. *Nat. Commun.* **8**, 1607. 10.1038/s41467-017-01792-x (2017). 29151575 10.1038/s41467-017-01792-x PMC5694765

[4] Challinor, A. J. et al. A meta-analysis of crop yield under climate change and adaptation. *Nat. Clim Change* **4**(4), 287 (2014).

[5] Taylor, C. & Schlenker, W. Environmental drivers of agricultural productivity growth: CO_2_ fertilization of US field crops. National Bureau of Economic Research Working paper 29320 (2023). http://www.nber.org/paper/w29320

[6] Forster, P. et al. The earth’s energy budget, climate feedbacks, and climate sensitivity. In *Climate Change 2021: The Physical Science Basis. Contribution of Working Group I to the Sixth Assessment Report of the Intergovernmental Panel on Climate Change* (eds Masson-Delmotte, V. et al.) 923–1054 (Cambridge University Press, Cambridge, 2021).

[7] https://gml.noaa.gov/webdata/ccgg/trends/co2/co2_annmean_mlo.txt

[8] Table 6.2 in Intergovernmental Panel on Climate Change (IPCC 2001) Radiative Forcing of Climate Change. Chapter 6 in the Third Assessment Report (*Climate Change 2001: The Scientific Basis* Chapter 6, (eds Houghton, J.T.,Y. et al.]. (Cambridge University Press, Cambridge, 2001).

[9] Meinshausen, M., Raper, S. C. B. & Wigley, T. M. L. (2011) Emulating coupled atmosphere-ocean and carbon cycle models with a simpler model, MAGICC6—part 1: Model description and calibration. *Atmos. Chem. Phys.* **11**, 1417–1456. 10.5194/acp-11-1417-2011 (2011).

[10] IPCC, 2021: Summary for Policymakers. In *Climate Change 2021: The Physical Science Basis. Contribution of Working Group I to the Sixth Assessment Report of the Intergovernmental Panel on Climate Change* (eds Masson-Delmotte, V. et al.] Table 2.1.

[11] R Core Team. *R: A Language and Environment for Statistical Computing* (R Foundation for Statistical Computing, Vienna, 2023).

[12] Blair, G., *et al*. Package ‘estimatr’ (2022). https://cran.r-project.org/package=estimatr Accessed June 2, 2013.

[13] McKitrick, R.Data and code to accompany "Extended Crop Yield Meta-analysis Data do not Support Upward SCC Revision"”, Mendeley Data, V1, 10.17632/w6rpyk3gyc.1 (2025). https://data.mendeley.com/datasets/w6rpyk3gyc/1 10.1038/s41598-025-90254-2 PMC11829979 39955458

