# Supplementary material for: Extended crop yield meta-analysis data do not support upward SCC revision
Source: Sci Rep. 2025 Feb 15;15:5575. doi: 10.1038/s41598-025-90254-2 (PMC11829979; doi:10.1038/s41598-025-90254-2)
Supplement: Supplementary file 1 — Supplementary Information. [file 41598_2025_90254_MOESM1_ESM.docx]

Online supplement for “Extended Crop Yield Meta-analysis Data do not Support Upward SCC Revision” by Ross McKitrick

Contents

[1. Notes on expansion of C14 data 1](#_Toc170142100)

[2. Results using different climate sensitivity and CO_2_ baseline assumptions 4](#_Toc170142101)

[3. Results by zone and C3/C4 distinction rather than crop type. 7](#_Toc170142102)

# 1. Notes on expansion of C14 data

Papers re-examined: Generally those for which CO_2_ was only variable missing. All entries refer to studies listed on pp. 18—29 of Challinor et al. 2014, Supplementary Information.

**Abou-Hadid** – couldn’t find source to confirm C14 entries.

**Alexandrov et al**: dC available in text for GCM sims. Couldn’t trace entries in C14 to source. Re-did based on those sims which were tied to specific GCMs. dP based on text discussion. Some C14 entries reported yield changes w/o CO_2_ change. C14 left out soybean results. New entries based on digitized results in Figs 6a-c, 8 records traceable to underlying GCMs, note 4.8C uses avg of Had&ECHAM.

**Arndt et al.** Experiment excludes effects of CO_2_ fertilization (p. 11) so dC=0

**Berg et al.** Uses A1B and A2 (SRES scenarios) compares 1970-1999 avg to 2070-2099 avg, so for each compare 2090 to 1990 to get dC from [IPCC TAR](https://www.ipcc.ch/site/assets/uploads/2018/03/TAR-APPENDICES.pdf) p. 807

**Brassard & Singh :**

- Cell I219 entered as -18.9 s/b -14.9
- I222 entered as -4.0 s/b-4.1
- Future CO_2_ levels shown in Table 3 but entered in wrong cell in spreadsheet table (col Z not F)

**Byjesh et al.** CO_2_ changes are described in paper but ambiguous and hard to match to experiments

**Calzadillah et al.** Uses B2 Scenario from TAR over span 2000-2050. CO_2_ increase not reported in paper but can be found in IPCC TAR WG1 Appendix. It is +109 ppm using ISAM, see [IPCC TAR](https://www.ipcc.ch/site/assets/uploads/2018/03/TAR-APPENDICES.pdf) p. 807

**Ciscar et al.** Note yield changes assigned from “Wheat, maize and soybeans” to Wheat only. Baseline is 1961-1990, for CO_2_ use 1980 = 337 per [IPCC TAR](https://www.ipcc.ch/site/assets/uploads/2018/03/TAR-APPENDICES.pdf) Appendix p. 807.

**El-Mayaar & Sonnentag** Base CO_2_ reported in C14 data base, changed CO_2_ =700ppm per Table 7 in paper.

**Giannakopoulos et al** - region not listed. Used NE/NW Mediterranean = Temp, SE/SW Mediterranean = Tropics

**Howden & Jones:** [online paper](http://www.cropscience.org.au/icsc2004/symposia/6/2/1848_howdensm.htm) contains none of the detailed yield changes shown in C14 file. Maybe C14 authors had SI from authors? Figures look like means, not medians since reported medians are close but not the same as C14 figures. CO_2_ changes use 370 ppm base and at 2070 sample from U[525,716] so change is U[155,346]. Since mean dC is same for all locations use midpoint = 250

**Iqbal** dC values were available in paper but not included in C14.

**Krishnan et al 2007**: many values not included. Table shows 5x5 dT, dC pairings but only 1^st^ and 5^th^ rows used, and dC values weren’t correctly coded. Also dP values were inferred from average GCM simulation using dC to interpolate from CO2 doubling experiment. Added about 50 records.

**Lakshari et al** supposedly study of 4 crops in India but the paper is 1 crop in Iran. CO2 multiplier = 1 but baseline not given so records coded as NA, but baseline supplied since it doesn’t matter. Source not found.

**Lobell et al (2008)** 94 obs and **Muller et al (2010)** 88 obs contain no entries for dT, dP or dC – why are they in the data base? Also **Peltonen-Sainio et al (2011)** 5 obs, **Ringler (2010)** 25 obs, others. 212 dY entries with no explanatory data!

**Osborne et al. (2012)** dC was stated in paper as 202 PPM but not included

**Reyenga et al.** dC values were available but not included

**Rozenzweig and Parry** dC values were available but not included

**Vesselin, A.** (undated) paper cited in spreadsheet but not listed in source list

**Walker and Schulze (2008)** Table 2 shows all experiment results but the yield changes don’t match tabulation in C14 so I re-entered them all. dC is increase from “current” climate to 555 ppm, midpoint of base case 1975-2005 is 1990 so using TAR Appendix that’s 353 ppm so dC=202. C14 appear to mistakenly have used doubling from 354.4 (~1990) but W&S used doubling from preindustrial to get endpoint of 555.

**Wang et al.** 27 entries in C14 correctly transcribed from Wang Table 4, but these are based on dC = 0 despite climate change. Wang et al Table 6 reports results with both dC=0 and dC>0, changes in CO_2_ from Table 5, dT and dP for Jilin from Table 1, same were used for adjacent regions of Changchun, Siping and Liaoyuan.

**Xiong et al. 2009** dC numbers obtained by using reported end CO_2_ values in Table 1 compared to C14-reported mid-baseline CO_2_ level.

# 2. Results using different climate sensitivity and CO_2_ baseline assumptions


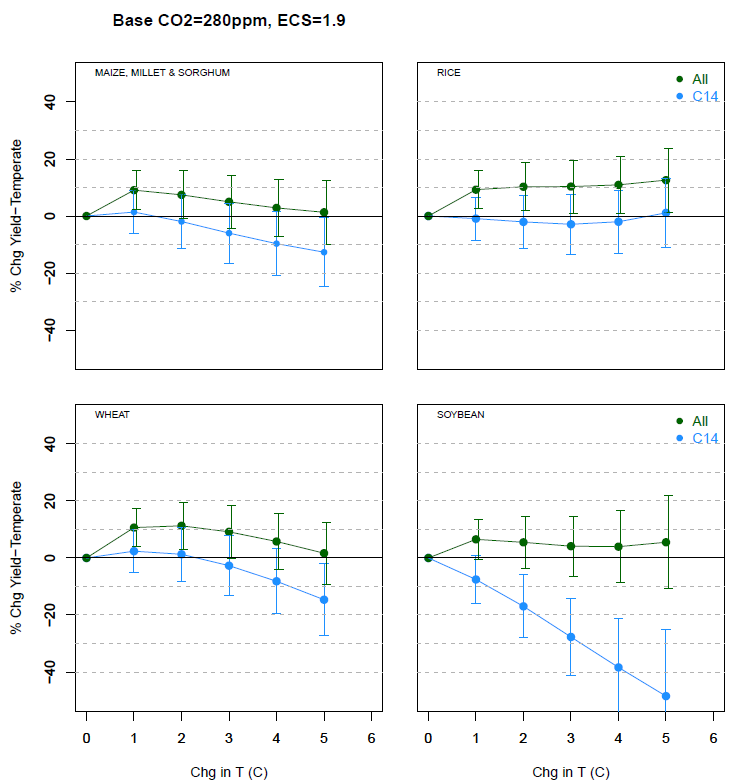


**Figure S1: Base CO_2_ = 280 ppm, ECS = 1.9K.**

**
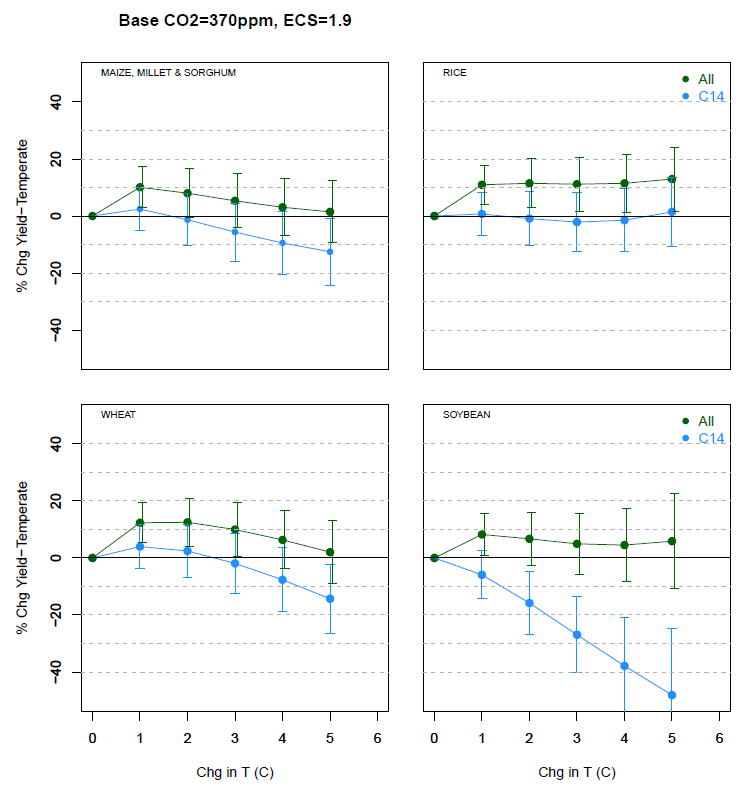
**

**Figure S2:** Base CO_2_ = 370 ppm, ECS=1.9K


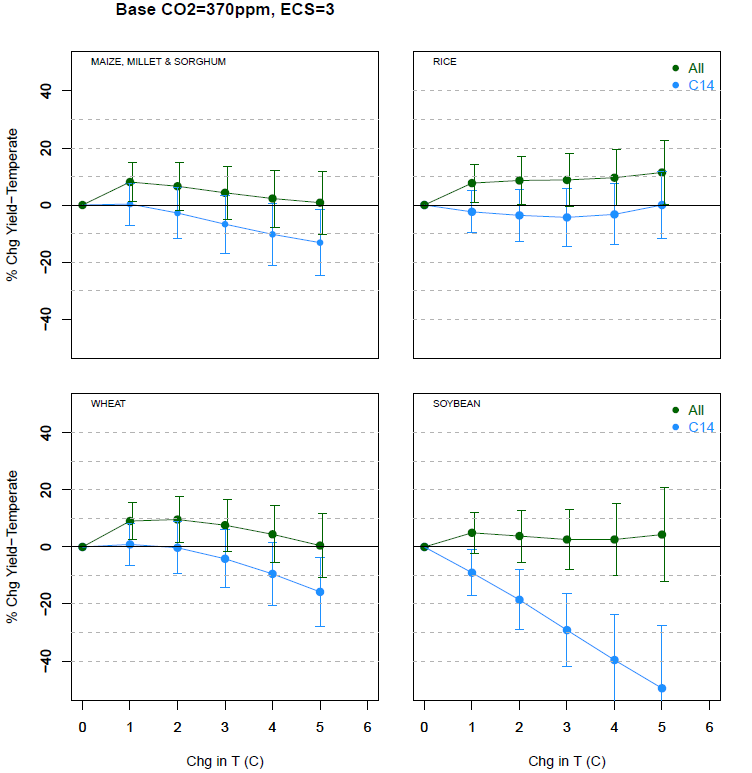


**Figure S3:** Base CO_2_ = 370 ppm, ECS=3.0K.

# 3. Results by zone and C3/C4 distinction rather than crop type.


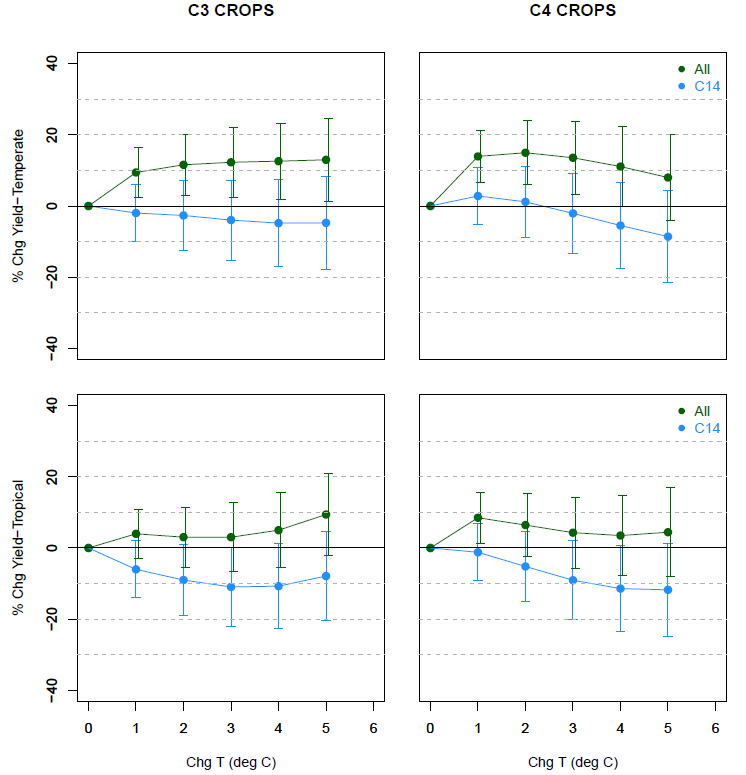


**Figure S4:** % yield changes versus change in temperature. Left column: C3 crops. Right column: C4 crops. Top row: Temperate zones. Bottom row: Tropical zones. Blue: C14 data. Green: Extended data set.
